# Spatiotemporal Distribution of the Magnitude of Completeness and b-Values in Mainland China Based on a Fused Multi-Source Earthquake Catalog

**DOI:** 10.3390/e27111137

**Published:** 2025-11-05

**Authors:** Chen Li, Ziyi Li, Mengqiao Duan, Lianqing Zhou

**Affiliations:** 1 Institute of Earthquake Forecasting, China Earthquake Administration, Beijing 100036, China; 2 Institute of Geophysics, China Earthquake Administration, Beijing 100081, China; 3 School of Earth and Space Sciences, Peking University, Beijing 100871, China

**Keywords:** earthquake catalog, magnitude of completeness, b-value, mainland China, statistical seismology, seismic monitoring, earthquake prediction

## Abstract

The b-value is a critical parameter for gauging seismic activity and is essential for seismic hazard assessment, monitoring stress evolution in focal zones, and forecasting major earthquakes. The minimum magnitude of completeness ($M_{c}$), a key indicator of the completeness of an earthquake catalog, reflects the monitoring capability of a seismic network and serves as a crucial foundation for the accurate calculation of the b-value. We began by integrating multi-source earthquake catalogs for mainland China using the nearest-neighbor method. Building on this, we employed a combination of partitioned time-series analysis and a grid-based spatial scanning technique to systematically investigate the spatiotemporal evolution of the $M_{c}$ and the b-value across mainland China and its adjacent regions. Our findings indicate the following: (1) Since the 1980s, the overall trend of $M_{c}$ has shifted from high and unstable values to low and stable ones. However, significant earthquake events can cause a notable short-term increase in the $M_{c}$. (2) The b-value exhibits strong fluctuations, primarily influenced by the dual effects of the tectonic stress field and catalog completeness. These fluctuations are particularly pronounced in highly active seismic regions such as the Sichuan–Yunnan area and Taiwan, whereas the western Tibetan Plateau has consistently maintained a low b-value. (3) The spatial distributions of both the $M_{c}$ and the b-value are markedly heterogeneous. By developing a unified and complete earthquake catalog for mainland China, our research highlights the qualitative leap in monitoring capabilities brought about by the continuous densification and technological upgrading of seismic networks. This dataset provides a solid foundation for future seismological research, disaster prevention practices, and especially for the development of AI-based earthquake prediction models.

## 1. Introduction

An earthquake catalog is a fundamental database that records essential information about seismic events, such as their origin time, epicenter location, magnitude, and focal depth. It is a core data source in seismological research. These catalogs are typically compiled chronologically and are essential for analyzing the spatiotemporal distribution of seismic activity. In China, the earthquake cataloging system is structured in a three-tiered hierarchy: provincial networks, the national network, and a unified national compilation center [1]. However, the completeness of these catalogs is significantly challenged by the uneven spatial distribution of seismic stations, disparities in monitoring capabilities, and inconsistencies in magnitude scaling methods. These issues result in discrepancies among catalogs compiled by different tiers and institutions, including missed events, duplicate entries, and magnitude biases. This discrepancies compromise the overall integrity and consistency of the data.

Earthquake catalogs are essential for seismic hazard assessment, lithospheric dynamics studies, earthquake-resistant engineering design, and seismic disaster mitigation, and they also provide the foundational data for earthquake prediction research. Earthquake catalog completeness directly impacts seismicity analysis, predictive model development, and disaster prevention strategy effectiveness. Seismicity parameters—such as the frequency–magnitude relationship, energy release characteristics, and b-value variations—are typically calculated from catalog data. These indicators can reveal short-term seismic activity and provide a basis for earthquake forecasting. Although a definitive mathematical or empirical relationship between these indicators and the time, location, and magnitude of future earthquakes has not yet been established, many studies are attempting to build predictive models using machine learning techniques based on these parameters. However, the efficacy and generalizability of these predictive methods remain highly dependent on the quality and integrity of the earthquake catalog. Therefore, establishing a complete and high-quality catalog is a fundamental prerequisite for investigating earthquake nucleation and rupture mechanics, developing statistical models, and ultimately, for advancing earthquake prediction [2,3,4,5,6,7].

To ensure catalog completeness, the scientific community has developed a variety of techniques. For instance, waveform matching, a highly sensitive event detection method, can identify microearthquakes and aftershocks within continuous waveform data that are often missed by conventional cataloging procedures. This approach significantly increases the detection rate of smaller seismic events. However, researchers often lack access to the raw waveform data required for such analysis, and catalogs from a single source frequently suffer from incomplete and inconsistent records, particularly concerning smaller earthquakes and aftershock sequences. Therefore, effectively integrating data from multiple catalogs is crucial for improving regional seismic completeness. Multi-source catalog integration methods, such as the improved nearest-neighbor algorithm, achieve this by establishing event correspondences across different datasets. By using a Euclidean metric that combines proximity in space, time, and magnitude, these methods automatically distinguish between duplicate and unique events, consolidate multiple catalogs, and enhance overall seismic record integrity and completeness [8]. Thus, effectively integrating data from multiple catalogs is crucial for improving regional aftershock sequences completeness.

The minimum magnitude of completeness ($M_{c}$) is a key metric for this purpose, representing the lowest magnitude at which all earthquakes within a specific space–time volume are reliably detected [9,10]. A catalog is considered “complete” only if all earthquakes with magnitudes greater than $M_{c}$ are captured within that window. Generally, the $M_{c}$ decreases over time due to technological advancements and increased station density. However, in the immediate aftermath a large earthquake, mainshock coda waves obscure smaller event signals, temporarily raising the $M_{c}$ [11]. Because seismic networks are often unevenly distributed, the $M_{c}$ can vary significantly across regions and time periods. Consequently, an accurate assessment of $M_{c}$ is critical for ensuring the overall integrity and reliability of an earthquake catalog. Current methods for estimating the $M_{c}$ fall into two broad categories: waveform-based and catalog-based approaches. Waveform-based methods evaluate the $M_{c}$ probabilistically by analyzing signal-to-noise ratios or phase-pick data, primarily to assess monitoring network performance [12,13]. Catalog-based statistical methods derive primarily from the Gutenberg–Richter frequency–magnitude relation [14], including the magnitude-order method [15], maximum curvature method [10], goodness-of-fit test [10], b-value stability method [16], Entire Magnitude Range technique [16,17], and Median-Based Analysis of the Segment Slope [18]. Among these approaches, stability-based methods, such as the CV stability approach, offer enhanced robustness. These methods systematically identify the magnitude range in which b-values remain stable [19,20]. This reduces the influence of short-term catalog fluctuations and improves the reliability of $M_{c}$ estimation. Each of these methods exhibits different sensitivities and is suited for different conditions, particularly when dealing with variations in sample size and spatiotemporal heterogeneity.

In seismicity analysis, the b-value quantifies the relative proportion of earthquakes of different magnitudes. It is a key parameter for measuring seismic activity and is closely correlated with crustal stress state, medium heterogeneity, and geothermal gradients [21,22,23,24,25].

A low b-value typically indicates high stress accumulation and is often associated with regions of elevated seismic hazard. Conversely, a high b-value suggests a greater proportion of smaller earthquakes and a more heterogeneous medium [24,26]. Consequently, the b-value has broad applications in seismic hazard assessment, focal zone stress evolution monitoring, and major earthquake forecasting.

B-value calculation methods have evolved from simple linear regression to sophisticated statistical approaches. The most widely used methods include the least squares method (LSM) [27], maximum likelihood estimation (MLE) [28,29,30], and the b-positive estimator [31,32]. Each method is suited to different scenarios: LSM is typically applied to regression analysis of large datasets, whereas MLE is ideal for smaller sample sizes and is particularly effective at minimizing the biasing influence of large earthquakes [28,33,34]. The b-positive estimator is valued for its robustness against the short-term catalog incompleteness that is characteristic of aftershock sequences. Van Der Elst [31] posits that the distribution of positive magnitude differences between successive earthquakes follows a Laplace distribution with the same b-value as the Gutenberg–Richter relation, but critically, without reference to a minimum magnitude of completeness. By analyzing only positive magnitude differences—where the second earthquake is larger than the first—this method minimizes bias from short-term aftershock incompleteness (STAI), as these differences are largely unaffected by the detection limitations imposed by preceding large events. Building upon this foundation, Lippiello and Petrillo [32] provided rigorous mathematical validation for the robustness of the b-positive estimator through conditional probability theory, demonstrating why it remains largely unaffected by detection issues. More importantly, they developed two enhanced variants: the “b-more-positive” estimator, which extends the analysis beyond consecutive earthquake pairs to include larger sets of positive magnitude differences, thereby improving statistical efficiency; and the “b-more-incomplete” estimator, which paradoxically achieves superior accuracy by artificially filtering the catalog to remove borderline detections near the completeness threshold. Their analysis revealed that the b-positive family of estimators performs optimally when catalogs exhibit sharper incompleteness transitions, leading to the counterintuitive conclusion that greater efficiency can be achieved with more incomplete but cleaner catalogs. These methodological refinements enable robust b-value monitoring throughout all phases of earthquake sequences without requiring explicit determination of time-varying completeness magnitudes.

In this study, we integrate earthquake data from multiple sources to construct a unified complete catalog, then systematically assess the minimum magnitude of completeness ($M_{c}$) across mainland China.

We adopt the analytical framework from Shi et al. (2020)’s work on the China Seismic Experimental Site (CSES) in the Sichuan–Yunnan region [35], employing a composite approach using three mainstream methods for evaluating $M_{c}$ spatiotemporal evolution: the magnitude-order method, maximum curvature method, and goodness-of-fit test. Based on this complete catalog, we calculate the b-value using the MLE and analyze its spatiotemporal variations, and then yield the changes in both the $M_{c}$ and the b-value for different regions and major active tectonic blocks over various time periods. Finally, we discuss the prospective applications for seismic hazard assessment and the development of predictive models, aiming to provide a solid data foundation for future seismological research and disaster mitigation practices, particularly for AI-based earthquake prediction models.

## 2. Data

### 2.1. Earthquake Catalog

**Figure 1:**
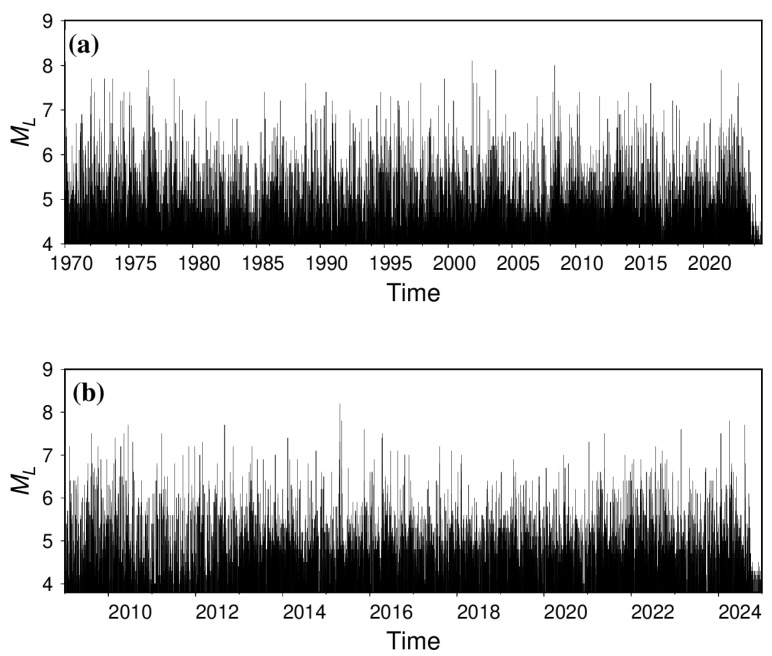
M-T diagrams for different catalogs. (**a**) CEIC_catalogue; (**b**) SMAC_catalogue.

We collected two earthquake catalog datasets to construct a comprehensive earthquake catalog dataset (Figure 1): the China Earthquake Information Center catalog (CEIC_catalogue, 1 January 1970 to 21 July 2024) and the catalog extracted from seismic phase reports provided by the Second Monitoring Center of the China Earthquake Administration (SMAC_catalogue, 1 January 2009 to 31 December 2024).

Considering the significant temporal heterogeneity between these catalogs and inherent observation systems differences across periods, we designed a hierarchical phase-merging strategy to construct a unified catalog with extended temporal coverage, high accuracy, and no internal redundancy. We selected overlapping observation periods across both catalogs, designating the CEIC catalog as the primary source while merging the supplementary catalog. During integration, we established a consistent standard reference framework within homogeneous subgroups. This approach effectively minimized potential systematic biases arising from combining datasets with vastly differing observational conditions, thereby ensuring the quality and completeness of the final synthetic catalog.

### 2.2. Earthquake Catalog Merge

Vorobieva et al. (2022) introduced a method for identifying and removing duplicate earthquake events when merging catalogs from different sources [8]. This approach utilizes a modified nearest-neighbor algorithm that calculates the Euclidean distance between events in the time–space domain to determine if they are duplicates.

The method employs a three-parameter model considering differences in origin time, longitude, and latitude, while intentionally excluding magnitude and depth. This decision was driven by practical data limitations: source depth is often missing from original catalogs or assigned a default value (e.g., 10 km), and magnitude is frequently reported using different scales, making direct comparisons unreliable.

The workflow proceeds as follows: one catalog is designated as primary, and the other as a supplementary catalog to be integrated. Assuming no duplicates exist within each individual catalog, the focus is on identifying redundant records referring to the same earthquake across both datasets. A proximity function is calculated for events pairs, and if this value falls below a predefined threshold, the pair is classified as a duplicate. Unmatched events in the supplementary catalog are considered unique and are merged into the primary catalog. This process can be iterated to progressively fuse multiple catalogs.

The proximity function is grounded in a probabilistic model, assuming that the discrepancies in detecting the same earthquake using different networks are random variables following a normal distribution, with a mean of zero for each parameter. This method significantly enhances the reliability of catalog merging, proving particularly effective in regions with high seismicity or complex earthquake sequences.

(1) $$f(∆T)=\frac{1}{\sigma_{T}\sqrt{2\pi }}exp(-\frac{{∆T}^{2}}{2\sigma_{T}^{2}})$$

(2) $$f(∆X)=\frac{1}{\sigma_{T}\sqrt{2\pi }}exp(-\frac{{∆X}^{2}}{2\sigma_{X}^{2}})$$

(3) $$f(∆Y)=\frac{1}{\sigma_{T}\sqrt{2\pi }}exp(-\frac{{∆Y}^{2}}{2\sigma_{Y}^{2}})$$

Here, ∆T, ∆X, and ∆Y represent the differences in origin time, longitude, and latitude between two event determinations, while $\sigma_{T}$, $\sigma_{X}$, and $\sigma_{Y}$ are the corresponding standard deviations. Vorobieva et al. (2022) [8] assume that all errors are independent.

Under this assumption, the joint probability density for a duplicate event is the product of the individual error probability densities for each parameter. This relationship follows a multivariate normal distribution, expressed as follows:

(4) $$f\left(\Delta T,\Delta X,\Delta Y\right)=\frac{1}{\sigma_{T}\sigma_{X}\sigma_{Y}{\left(2\pi \right)}^{3/2}}exp\left(-\frac{1}{2}\left(\frac{\Delta T^{2}}{\sigma_{T}^{2}}+\frac{\Delta X^{2}}{\sigma_{X}^{2}}+\frac{\Delta Y^{2}}{\sigma_{Y}^{2}}\right)\right)$$

The Euclidean metric is a natural derivation from the relationship above.

(5) $$R=\sqrt{\frac{\Delta T^{2}}{\sigma_{T}^{2}}+\frac{\Delta X^{2}}{\sigma_{X}^{2}}+\frac{\Delta Y^{2}}{\sigma_{Y}^{2}}}$$

**Figure 2:**
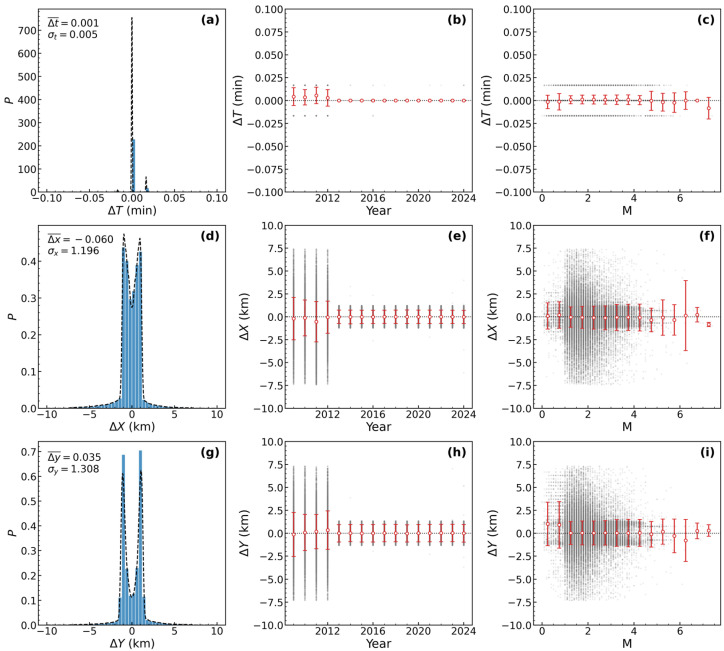
Distribution and stability analysis of parameter differences for potential duplicate events. The first column (**a**,**d**,**g**) displays histograms of the differences in origin ∆T, ∆X, and ∆Y between nearest-neighbor events in the CEIC and SMAC catalogs. The y-axis represents the number of event pairs (count) for each bin. The second and third columns (**b**,**c**,**e**,**f**,**h**,**i**) show the variation in these differences as a function of event year and magnitude, respectively. In these plots, the red dots represent the mean difference, and the error bars denote the corresponding standard deviation.

We designated the CEIC catalog as the primary and the SMAC catalog as the supplementary catalog. For initial duplicate events identification, we accounted for progressive improvements in seismic monitoring network capabilities since 2009. Accordingly, we set the temporal standard deviation to $\sigma_{T}=0.1\;min$ and the spatial standard deviations to $\sigma_{X}=\sigma_{Y}=15\;km$ for the merging process. To validate the probabilistic assumptions underlying our method and to determine the optimal parameters for the distance metric (Equation (1)), we conducted a detailed statistical analysis of the differences in origin time (∆T), longitude (∆X), and latitude (∆Y) between potential duplicate event pairs. The results are presented in Figure 2.

Following the removal of definitive duplicates, we conducted a further statistical analysis of the differences in origin time (∆T), longitude (∆X), and latitude (∆Y). The results, shown in Figure 2, reveal a high degree of consistency between the CEIC and SMAC catalogs. The most striking feature is the distribution of the origin time difference (∆T). Rather than following a normal distribution, it manifests as an impulse function at ∆T=0. This indicates that potential duplicate events in the two catalogs share identical recorded origin times, making the effective standard deviation $\sigma_{T}=0$. In contrast, the distributions for the longitude difference (∆X) and latitude difference (∆Y) exhibit clear, zero-centered, and approximately Gaussian (normal) forms. The mean differences are negligible ($\bar{∆X}=-0.06\;km$ and $\bar{∆Y}=0.035\;km$), which suggests the presence of minor random errors but no significant systematic bias in event location between the two catalogs. Based on these distributions, we derived the following optimized standard deviation parameters for our subsequent analysis:$\;\sigma_{T}=0.05\;min$,$\;\sigma_{X}=1.196,$ and $\sigma_{Y}=1.308\;km$.

Furthermore, we analyzed whether these parameter differences vary with time or earthquake magnitude (Figure 2). On a macroscopic level, both the mean differences (red dots) and their scatter (error bars) remain broadly stable, exhibiting no clear systematic trends either over time or with increasing magnitude. This result confirms that the statistical characteristics of the errors are fundamentally consistent, providing a sound justification for using a single, fixed set of σ parameters for the subsequent merger of the entire catalog.

However, closer inspection reveals two important details. First, in the third column of Figure 2 (particularly for ∆X and ∆Y), there is a clear trend of greater scatter in the location differences for smaller-magnitude earthquakes. This observation is consistent with the fundamental principles of seismic locating: smaller events produce weaker signals with lower signal-to-noise ratios. This makes their calculated locations more susceptible to factors like uneven station distribution and background noise—especially in areas with sparse network coverage—resulting in larger location errors. Conversely, as magnitude increases, seismic signals become clearer and are recorded by more distant stations, which improves location precision and leads to smaller discrepancies.

Second, the data points prior to 2013 exhibit noticeably greater scatter compared to those from later years. This likely indicates that an increase in network density improved the azimuthal coverage of seismic sources, leading to the higher consistency and smaller random errors observed in the post-2013 data.

**Figure 3:**
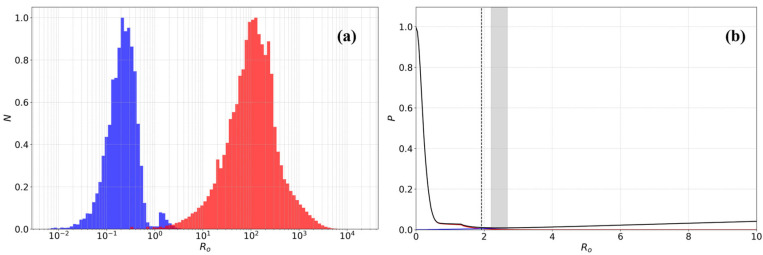
R_0_ distribution and classification error analysis. (**a**) Comparison of the R_0_ metric distribution for cross-catalog (CEIC/SMAC, blue histogram) versus within-catalog (CEIC/CEIC, red histogram) nearest-neighbor pairs. (**b**) Classification error rates as a function of the R_0_ threshold. The red, blue, and black curves show the false negative rate (Type I), false positive rate (Type II), and total error rate, respectively. The gray bar highlights the region of minimum total error (approx. 0.5%).

Utilizing the previously optimized standard deviation parameters, we calculated and analyzed the probability density distribution of R_0_ to evaluate its effectiveness in identifying duplicate events and to determine an optimal classification threshold. Figure 3a contrasts two key distributions. The R_0_ distribution for cross-catalog nearest-neighbor pairs (blue histogram) exhibits a distinct, concentrated peak at low values (R_0_ < 1). This peak represents the population of true duplicate events, which are highly correlated in space and time. In stark contrast, the R_0_ distribution for nearest-neighbor pairs within the CEIC catalog (red histogram) characterizes the random proximity inherent in background seismicity. The clear separation between these two distributions empirically confirms that the R_0_ metric can effectively distinguish the signal (true duplicates) from the noise (randomly adjacent events).

Building on this, we optimized the R_0_ threshold by quantitatively analyzing the classification error rates (Figure 3b). This process involved balancing two types of errors: Type I error (the false negative rate, red curve), which is the probability of failing to identify a true duplicate event; and Type II error (the false positive rate, blue curve), which is the probability of incorrectly classifying a distinct, nearby event as a duplicate. This rate was estimated from the R_0_ distribution of events within the CEIC catalog. As shown in the figure, the total error rate trend (black curve) indicates that the combined classification error is minimized when the threshold is set to R_0_ = 1.9.

## 3. Methodology

### 3.1. Calculation of Minimum Completeness Magnitude

The monitoring capability of a seismic network is not constant across vast geographical areas and multi-decade time spans. In regions with dense station coverage and low background noise, numerous microearthquakes can be comprehensively recorded. Conversely, in areas with sparse instrumentation or complex environments, many smaller events are likely missed, leading to catalog incompleteness. This spatiotemporal inconsistency in detection capability directly impacts seismicity levels analysis. Therefore, before undertaking quantitative seismicity analysis or hazard assessment, scientific evaluation of the catalog completeness is essential and involves precisely determining the minimum magnitude of completeness ($M_{c}$) for different spatiotemporal domains to ensure data reliability and consistency. Even a minor deviation in the $M_{c}$ value can cause dramatic change in the number of earthquakes included in the analysis, directly affect the calculation of seismicity parameters, such as the b-value, and ultimately have a decisive impact on the reliability of the final conclusions.

#### 3.1.1. Magnitude-Index Method

The Magnitude-Index Method is a qualitative approach used to analyze the temporal evolution of the $M_{c}$. The procedure begins by indexing earthquakes chronologically and dividing them into groups of equal size. For each group, the number of events within various magnitude bins is counted, and this frequency information is then visualized to illustrate the time-series evolution of the $M_{c}$ [9,28]. Compared to traditional magnitude–time plots, the Magnitude-Index Method not only effectively mitigates distortions caused by high-rate event clusters like aftershocks and swarms—thereby improving observational clarity—but also exhibits sensitivity to short-term changes in seismic network monitoring capability.

#### 3.1.2. Maximum Curvature

The Maximum Curvature (MAXC) method is a rapid technique for calculating the $M_{c}$ that does not require a priori parameters [10,23]. This approach determines the $M_{c}$ by taking the first derivative of the cumulative frequency–magnitude distribution (FMD) and finding the magnitude that corresponds to its maximum value. For the non-cumulative FMD, the $M_{c}$ is simply defined as the magnitude bin with the highest count of recorded earthquakes [16].

#### 3.1.3. Goodness-of-Fit

The goodness-of-fit (GFT) method calculates $M_{c}$ by comparing the observed cumulative frequency–magnitude distribution (FMD) from the earthquake catalog with the theoretical curve fitted by the Gutenberg–Richter (G-R) relation. For each candidate cutoff magnitude, $M_{i}$, the maximum likelihood method is used to estimate the G-R parameters, $a_{i}$ and $b_{i}$, and generate a corresponding fitted curve. The goodness-of-fit, R, is then given by the following expression:

(6) $$R\left(a_{i},b_{i},M_{i}\right)=1-\frac{\sum_{M_{k}=M_{i}}^{{M_{k}=M}_{max}}{|B}_{k}-S_{k}\left(a_{i},b_{i},M_{i}\right)|}{\sum_{M_{k}=M_{i}}^{{M_{k}=M}_{max}}B_{k}}$$

where $B_{k}$ represents the cumulative count of earthquakes observed in the magnitude bin $M_{k}$ (for all bins between the cutoff magnitude $M_{i}$ and the maximum magnitude $M_{max}$);

$S_{k}\left(a_{i},b_{i},M_{i}\right)$ is the corresponding theoretical value estimated from the fitted Gutenberg–Richter (G-R) relation:

(7) $$S_{k}\left(a_{i},b_{i},M_{i}\right)=10^{\;a_{i}-b_{i}M_{i}}$$

$M_{c}$ is typically taken as the first cutoff magnitude $M_{i}$, for which *R* reaches a predefined goodness-of-fit threshold (e.g., 90% or 95%), or as the $M_{i}$ that corresponds to the minimum value of *R*.

### 3.2. Calculation of b-Value

The choice of calculation method is critical to b-value accuracy. While the traditional least-squares method is straightforward, it can introduce systematic bias by assigning different weights to earthquakes of different magnitudes [36]. In contrast, the maximum likelihood estimation (MLE) method assigns equal weight to every event in the catalog and is known to produce smaller errors, be less computationally intensive, and yield more stable results, particularly with small sample sizes [20,25]. Therefore, to ensure the accuracy and reliability of our b-value calculations, this study employs the MLE method. Following the classic approach proposed by Aki (1965) [28], once the minimum magnitude of completeness ($M_{c}$) has been determined, the b-value is calculated using the following formula:

(8) $$b=\frac{\log_{10}\left(e\right)}{\bar{M}-\left(M_{c}-\frac{\Delta M}{2}\right)}$$

In this equation, $\bar{M}$ represents the mean magnitude, $M_{c}$ is the minimum magnitude of completeness, *e* is the base of the natural logarithm, and ΔM is the magnitude bin width.

### 3.3. Integrated Assessment Analyses

Building upon the qualitative analysis of the 1970–2024 earthquake catalog for mainland China from the Magnitude-Index Method, we calculate the $M_{c}$ in both temporal and spatial domains using a sliding window approach. Specifically, we employ a hybrid method that combines the MAXC and GFT techniques to obtain both $M_{c}$ and its uncertainty, $\delta M_{c}$.

First, we determine an optimal estimate, $M_{c}^{\mathrm{best}}$, by applying a prioritized rule to three candidate values: $M_{c}$ from MAXC ($M_{c}^{MAXC}$) and $M_{c}$ values from GFT at 95% and 90% goodness-of-fit levels ($M_{c}^{95}$ and $M_{c}^{90}$, respectively). The selection hierarchy is $M_{c}^{95}\to M_{c}^{90}\to M_{c}^{MAXC}$. This procedure balances the reliability of the GFT method with the stability of the MAXC method and provides a solid basis for subsequent uncertainty estimation. The specific rules are as follows:

- If the GFT goodness-of-fit is ≥95%, then $M_{c}^{best}=M_{c}^{95}$.
- If 90% ≤ GFT goodness-of-fit < 95%, then $M_{c}^{best}=M_{c}^{90}$.
- If the GFT goodness-of-fit is <90%, then $M_{c}^{best}=M_{c}^{MAXC}$.

Next, we use a Monte Carlo approximation via the bootstrap method [37] to calculate the final $M_{c}$ and its uncertainty, $\delta M_{c}$. This involves creating numerous bootstrap catalogs by randomly sampling the original earthquakes set with replacement. For each bootstrap sample, we re-calculate $M_{c}^{best}$ using the same combined method described above. The mean of the resulting $M_{c}^{best}$ distribution values is taken as the final $M_{c}$, and the standard deviation is taken as its uncertainty, $\delta M_{c}$. The formula is as follows:

(9) $$M_{c}=\bar{M_{c}^{best}}=\frac{1}{B}\sum_{b=1}^{B}{M_{c}^{best}}_{b}$$

(10) $$\delta M_{c}=\sqrt{\frac{1}{B-1}\sum_{b=1}^{B}{\left({M_{c}^{best}}_{b}-\bar{M_{c}^{\mathrm{best}}}\right)}^{2}}$$

Previous studies have shown that results stabilize when the number of bootstrap iterations (B) reaches 200 [16,35]. Therefore, our quantitative analysis employs 200 bootstrap resamples to calculate the $M_{c}$ and its uncertainty, $\delta M_{c}$. As a quality control measure, we only retain results where $\delta M_{c}\leq 0.4$; any calculation yielding uncertainty greater than this threshold is discarded. Following this procedure, we systematically compute $M_{c}$ and $\delta M_{c}$ for each temporal and spatial window.

Given that b-value calculations are highly sensitive to catalog completeness, we implement a crucial subsequent step to ensure stable and reliable results. For every data window with a successfully determined $M_{c}$, we use that value to truncate the catalog, retaining only events with magnitudes greater than or equal to the $M_{c}$. The b-value is then calculated for this complete catalog subset using the maximum likelihood estimation method.

### 3.4. Regional Division

**Figure 4:**
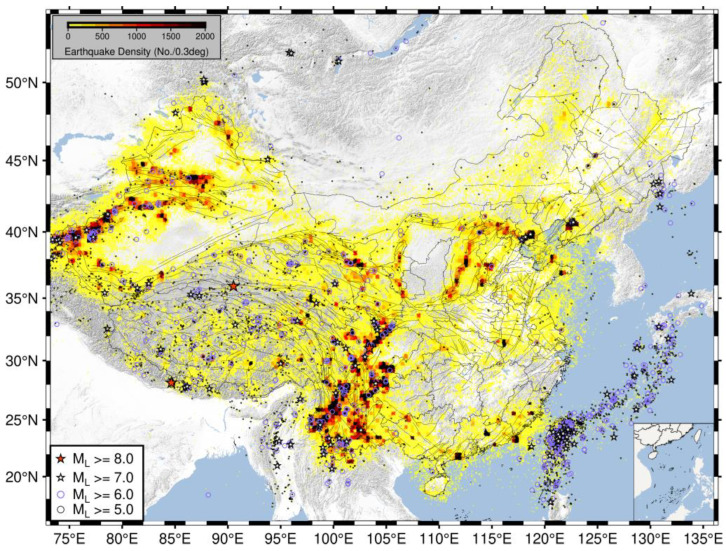
Spatial distribution and density of seismicity in mainland China. The map illustrates the spatial density of earthquakes, where the yellow color gradient quantifies the number of events occurring within each 0.3-degree grid cell.

We first generated a spatial density map of seismic activity using the unified 1970–2024 earthquake catalog to provide a macroscopic overview of seismicity patterns across mainland China (Figure 4). The map clearly reveals that seismic activity in China exhibits significant spatial non-uniformity. Earthquakes are highly concentrated in western regions, forming several high-density bands, particularly along the North–South Seismic Belt, within the Tibetan Plateau and its margins, and across the Tianshan seismic belt in Xinjiang. In contrast, seismicity density is markedly lower throughout the vast eastern and northeastern regions. Furthermore, the moderate-to-strong earthquakes indicated on the map occur predominantly within or along the edges of these high-density zones, further corroborating the seismic hazard posed by these tectonically active belts.

To perform meaningful analysis of $M_{c}$ and b-value evolution across mainland China, we first partitioned the study area into smaller, tectonically distinct regions. This regionalization is critical because a single, nationwide analysis would be misleading. China’s vast scale and complex geology mean that a monolithic approach would average out significant local variations in seismicity, leading to biased and uninformative results.

**Figure 5:**
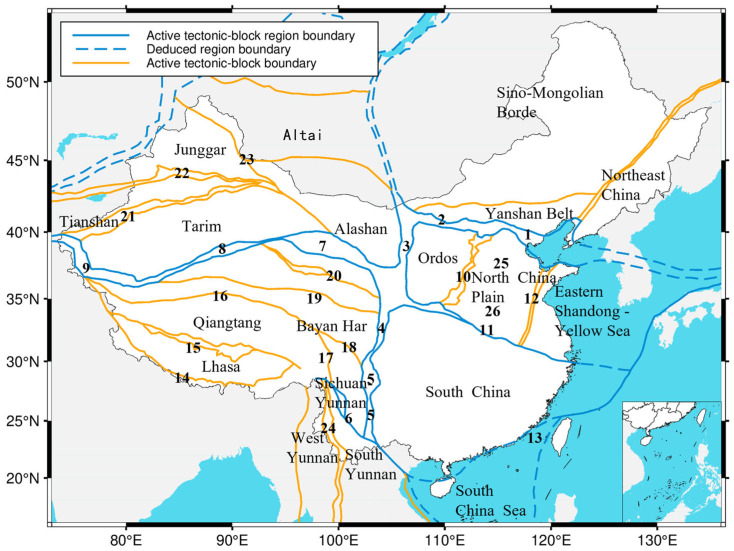
Division of active tectonic blocks and distribution of their boundary zones in mainland China (modified from [38]). Numbers 1–26 indicate the active block boundary IDs as listed in Table 1 (No. column).

Our regionalization scheme is based on the well-established, two-tiered active block model of Zhang et al. (2005) [38], which divides mainland China into six Level I and 22 Level II blocks grounded in the country’s fundamental tectonic and seismicity patterns. Crucially, the boundaries between these blocks are not arbitrary lines; they are the primary zones of stress accumulation and differential crustal movement. As such, they host the vast majority of strong earthquakes and define the most significant seismic hazard belts in the nation (Table 1 and Figure 5).

**Figure 6:**
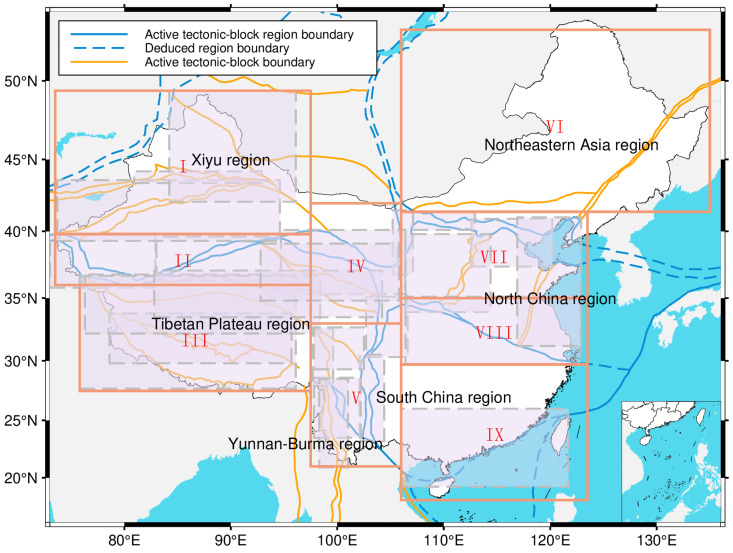
Map of regional divisions and active block boundary zones. Orange boxes delineate the final study regions and pink-shaded areas indicate the distribution of active block boundary zones. Roman numerals I–IX correspond to zone IDs in Table 2 (No. column).

We defined the final nine study regions by overlaying our seismicity density map onto the established framework of active tectonic blocks and their boundaries (Table 2 and Figure 6). This data-driven step was critical for resolving minor overlaps in the tectonic zones and ensuring our partitions reflect the actual spatial distribution of recent earthquakes. This integration of tectonic theory with empirical data yields a robust regionalization that captures the unique seismicity characteristics of each block and establishes a reliable foundation for our spatiotemporal analysis of $M_{c}$ and b-values.

**Figure 7:**
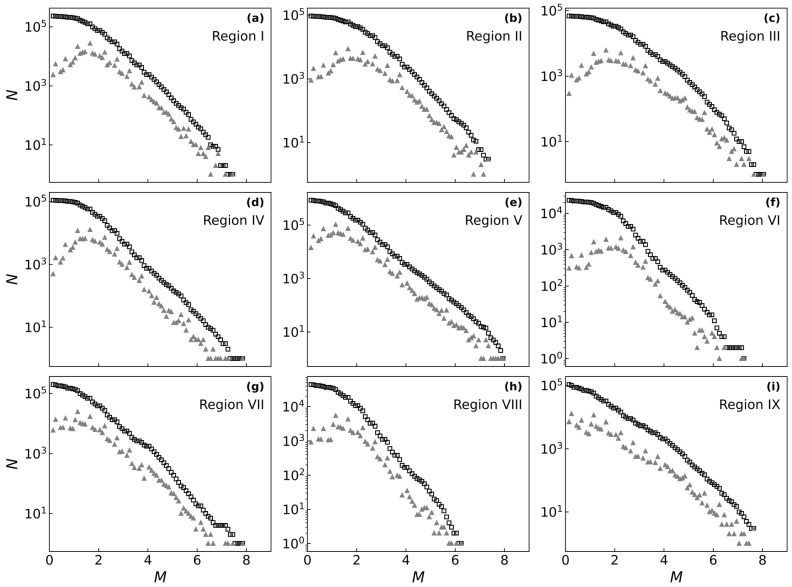
Frequency–magnitude distributions (FMDs). FMDs for the nine sub-regions of mainland China from 1970 to 2024. Open black squares represent the cumulative number of events, while gray triangles show the non-cumulative, binned frequency. The y-axis is on a logarithmic scale, and the x-axis represents magnitude. Subplots (**a**–**i**) show the results for seismic Regions I–IX, respectively, as defined in Figure 6 and listed in Table 2.

The frequency–magnitude distributions across the nine regions (Figure 7) generally exhibits a power-law decay consistent with the Gutenberg–Richter law, characterized by a higher frequency of low-magnitude events and a lower frequency of high-magnitude events. The cumulative curves for these distributions approximate a straight line in the intermediate magnitude range. At the extremes of the distribution, several features are apparent. The peak of the non-cumulative curve at the low-magnitude end reveals a regional catalog completeness threshold ($M_{c}$) of approximately magnitude 1 to 2. At the high-magnitude end, the scarcity of large earthquake samples results in significant data point dispersion, which compromises the stability of the statistical fit.

Although this general pattern is common across all zones, the seismicity levels and energy release patterns vary significantly among the regions. In terms of total event count, Region V demonstrates the highest activity, with nearly 10^5^ events, followed by Region VI and Region VIII (approximately 10^4^ events). Conversely, Regions III and Region IX exhibit the lowest activity levels; their sparse event counts exacerbate the uncertainty of fitting the distribution at the high-magnitude end. Furthermore, the b-value, which is the slope of the cumulative curve and reflects the relative proportion of large to small earthquakes, also differs by region. Region VII displays the shallowest slope (a low b-value), indicating a relatively higher proportion of large earthquakes. In sharp contrast, Regions I, II, and IV have steep slopes (high b-values), suggesting that energy is predominantly released through a multitude of small earthquakes. Meanwhile, regions such as V, VI, and VIII show moderate b-values, representing a more typical energy release pattern.

## 4. Result

To facilitate qualitative analysis of the magnitude of completeness ($M_{c}$), we generated a magnitude-sequence plot for the entire mainland China catalog. Based on the data volume and the approximate $M_{c}$ levels revealed in this plot, we segmented the earthquake catalog into distinct time periods. Since the 1970s, seismic monitoring capabilities in mainland China have undergone significant enhancement, clearly reflected in the magnitude-sequence plot. The color variations in the plot represent the density of seismic events over time, with brighter bands indicating higher frequencies of seismic activity.

Initially, from 1970 to 1985, the plot indicates an average $M_{c}$ of approximately 2.3. During this era, records of earthquakes below M 2.0 were sparse, suggesting that the monitoring system was still developing and unable to capture many smaller events. With subsequent technological advancements between 1985 and 2001, the $M_{c}$ gradually decreased to 1.6. A persistent high-density band emerged around 2.5, marking improved coverage of lower-magnitude events. From 2001 to 2009, the $M_{c}$ decreased further to 1.4 as the number of recorded low-magnitude earthquakes increased, reflecting notable improvement in monitoring capabilities and the progressive capture of microearthquakes.

After 2009, following progressive enhancement of the national seismic network, the $M_{c}$ dropped to 1.1 and has stabilized since 2017, demonstrating the comprehensive coverage and stability of the nationwide network. Over the last decade, the $M_{c}$ value has approached 0.9~1.0. Although the catalog for microearthquakes below 0.5 remains incomplete, overall monitoring capability has reached a very high level. This progression demonstrates that over the past half-century, China’s seismic monitoring system has made substantial strides in both technology and data integrity, achieving overall catalog completeness improvement of approximately 1.5 to 1.7 magnitude units.

**Figure 8:**
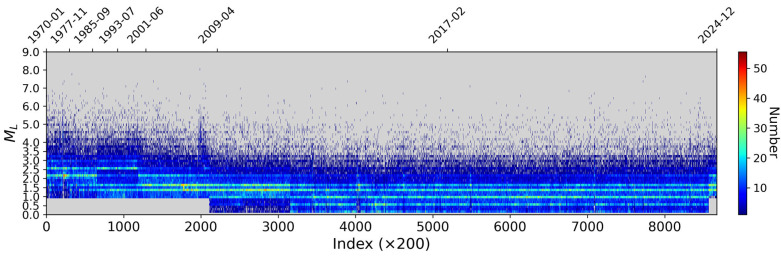
Magnitude-sequence diagram for mainland China (1970–2024).

Using the magnitude-sequence method (Figure 8), we identified major shifts in both $M_{c}$ and catalog size for mainland China occurring around 1987, 2000, and 2009. Based on these transition points, we divided the catalog into four periods: 1970–1987, 1987–2000, 2000–2009, and 2009–2024.

We then calculated the temporal evolution of the $M_{c}$ across the entire study area. To adapt to the increasing number of recorded events over time, we employed a moving-window analysis with period-specific parameters. For the 1970–1987 period, we used a window of 1800 events and a step of 800. For 1987–2000, the window and step were 2000 and 1000 events, respectively. For 2000–2009, these parameters increased to 4000 and 1500, and for 2009–2024, to 8000 and 4000. The resulting time series is plotted below.

**Figure 9:**
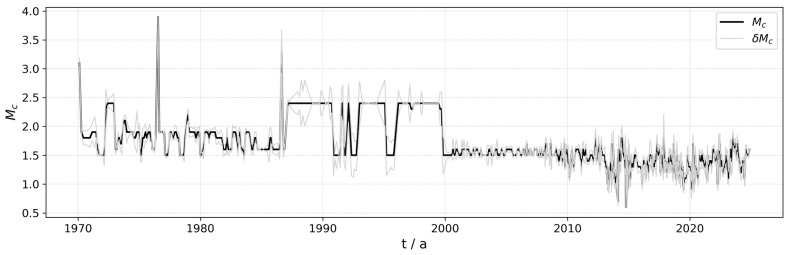
$M_{c}-t$ plot for mainland China, 1970–2024.

Figure 9 illustrates the temporal evolution of the $M_{c}$ for mainland China from 1970 to 2024. As shown in the plot, the $M_{c}$ exhibited significant fluctuations during the 1970s and early 1980s, marked by several sharp peaks that reflect the technical instability of the seismic monitoring network during its initial development. Beginning in the 1990s, the $M_{c}$ began a gradual decline, eventually stabilizing at approximately 1.5 after the year 2000, indicating enhanced monitoring capabilities. Post 2010, the $M_{c}$ value has remained stable within the 1.0 to 2.0 range, demonstrating that China’s seismic network and technology have matured, allowing for the effective recording and monitoring of smaller-magnitude events. The variations in standard deviation (${\delta M}_{c}$) mirror $M_{c}$ behavior. Periods of high standard deviation typically coincide with sharp fluctuations in the $M_{c}$, suggesting lower data stability during those times. Overall, the steady decrease in the $M_{c}$ and the concurrent reduction in its standard deviation signify progressive improvement and stabilization of the seismic monitoring system over the past several decades.

### 4.1. Analysis of the Time-Variance of $M_{c}$ and b-Values

**Figure 10:**
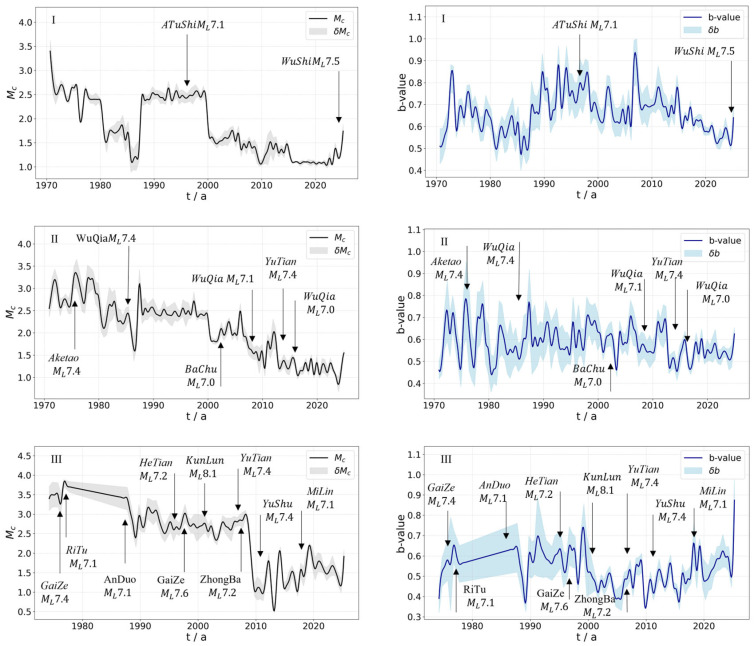

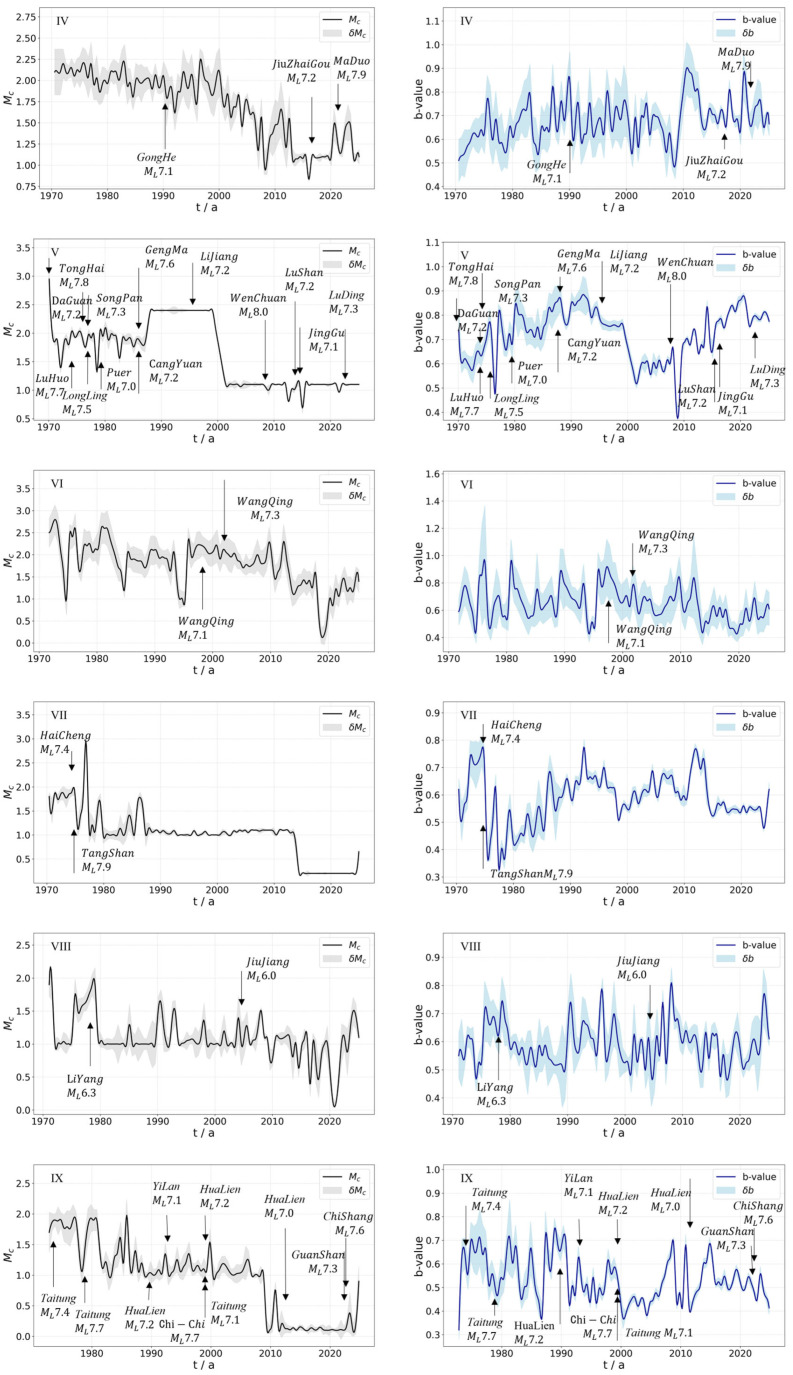
$M_{c}-t$ and b−t plots for different seismic zones, 1970–2024. Roman numerals I–IX indicate the seismic zone IDs as shown in Figure 6 and detailed in Table 2.

We identified significant spatiotemporal heterogeneity in the evolution of these parameters across different regions. This prompted detailed analysis of the temporal evolution of the $M_{c}$ and b-values for each zone, using region-specific parameters (Figure 10). At the regional scale, the $M_{c}$ values across mainland China generally transitioned from high and unstable values to low and stable values. In contrast, b-values in each region exhibited considerable fluctuations throughout the entire period, commonly characterized by episodic fluctuations with alternating peaks and troughs. Overall, they did not exhibit sustained, long-term monotonic increases or decreases.

Specifically, in Region I, the $M_{c}$ remained between 2.5 and 3.0 before 1980 and exhibited some volatility. After 1980, the $M_{c}$ experienced more pronounced fluctuations but gradually stabilized at around 2.5 after 1990. After 2000, the $M_{c}$ decreased significantly and was maintained at a stable level between 1.0 and 1.5 for an extended period. However, after 2020, the $M_{c}$ rose noticeably, with this upward trend becoming more pronounced following the $M_{L}$7.5 Wushi earthquake in 2024. The b-value in this region generally varied between 0.5 and 1.0. In the early 1970s, it rose sharply to about 0.85 before dropping rapidly to 0.6. Despite a brief recovery, it showed a general downward trend with fluctuations until 1987. Between 1990 and 2010, the b-value remained between 0.6 and 0.8. A significant drop occurred in 2010, followed by a continued decrease until signs of recovery appeared in 2024.

In Region II, the $M_{c}$ showed large-amplitude fluctuations between 1970 and 1990, closely correlated with several strong earthquakes in years such as 1974 and 1985. From 1990 to 2000, the $M_{c}$ was stable at approximately 2.5. After 2000, it gradually decreased and has since remained between 1.5 and 2.0. The b-value experienced high-amplitude oscillations between 1970 and 1985, varying from approximately 0.4 to 0.8. From 1985 to 2010, oscillation amplitudes diminished, with values primarily between 0.55 and 0.7. Since 2010, b-values have remained stable, below 0.6.

In Region III, the $M_{c}$ remained at a high level of 3.5 to 4.0 between 1975 and 1990, influenced by multiple strong earthquakes. After 1990, the $M_{c}$ dropped to between 2.0 and 2.5, decreasing further to the 1.0 to 2.0 since 2004. The b-value in this region fluctuated within a narrow range at low levels between 1970 and 1990, showing an inverse trend to the $M_{c}$ as it gradually rose from 0.4 to 0.65. Around 1989, the b-value experienced an abrupt drop but quickly rebounded, remaining between 0.5 and 0.7 around the year 2000. Since 2000, the b-value has exhibited a gradual upward trend.

In Region IV, the $M_{c}$ generally remained between 1.8 and 2.25 from 1970 to 2000, with clear overall fluctuations. Between 2000 and 2010, the $M_{c}$ gradually decreased to 1.0, and after 2010, remained between 1.0 and 1.75. Several major earthquakes in this region significantly impacted the $M_{c}$; for instance, both the 1990 Gonghe earthquake and the 2017 Jiuzhaigou earthquake caused the $M_{c}$ to initially decrease before rising, while the 2021 $M_{L}$7.9 Maduo, Qinghai earthquake led to a subsequent increase. The b-value gradually rose to 0.75 from 1970 to 1976. Between 1976 and 2009, it oscillated primarily between 0.5 and 0.8, marked by occasional prominent peaks and troughs. After 2009, the b-value began rising toward 0.9, then slowly decreased, and has since fluctuated between 0.6 and 0.8.

Region V, covering the border of Sichuan and Gansu provinces down to southern Yunnan, is tectonically complex and characterized by frequent and intense seismicity. In the early 1970s, following the Tonghai earthquake, the $M_{c}$ began to decline gradually from 3.0. Between 1970 and 1989, the $M_{c}$ showed considerable fluctuation, influenced by multiple strong events including the 1973 Luhuo, 1974 Daguan, 1976 Longling, and 1988 Gengma earthquakes. From 1989 to 2000, the $M_{c}$ was relatively stable at around 2.4. During the 2000–2024 period, the $M_{c}$ dropped markedly to 1.2, experiencing only brief perturbations during major earthquakes such as the 2008 Wenchuan, 2013 Lushan, 2014 Jinggu, and 2022 Luding events, followed by rapid recovery. The b-value showed an upward trend with significant oscillations from 1970 to 1992, ranging from approximately 0.5 to 0.9. It remained between 0.7 and 0.9 from 1992 to 2000, then decreased and stabilized at 0.5~0.65 from 2000 to 2008. During the Wenchuan earthquake, the b-value saw a sharp drop followed by a rapid rebound. Since 2010, the b-value has remained at a relatively high level, between approximately 0.65 and 0.9.

In Region VI, the $M_{c}$ started at approximately 2.2 in 1972, first decreasing to 1.0 before rapidly rebounding to 2.6. It then entered a long fluctuation period. From 1980 to 2012, the $M_{c}$ remained between 1.5 and 2.5, and from 2012 to 2024, it decreased further, remaining stable between 1.0 and 1.5. The b-value lacked a monotonic trend and was instead characterized by episodic fluctuations, ranging between 0.4 and 1.0.

In Region VII, the $M_{c}$ exhibited a trend of first increasing and then decreasing. Between 1970 and 1976, it was between 1.5 and 2.2. From 1977 to 1983, fluctuations intensified; influenced by the 1975 Haicheng and 1976 Tangshan earthquakes, the $M_{c}$ spiked to its peak value (slightly above 3.0), ranging from 2.5 to 3.2. From 1984 to 2013, it gradually decreased and stabilized around 1.0~1.5, and from 2013 to 2020, it dropped to 0.8~1.0. The b-value in this region rose from 1970 to 1975. Following a drop to 0.38 due to a strong earthquake in 1975, it quickly rebounded to 0.65. It increased annually from 1976 to 1990, staying between 0.4 and 0.7, and has been relatively stable between 0.5 and 0.8 since 1990.

In Region VIII, the $M_{c}$ showed a slow, long-term decreasing trend punctuated by multiple fluctuations. It experienced episodic high values and sharp peaks between 2.0 and 2.5 from 1970 to the early 1980s. From 1980 to 1990, it oscillated between 1.0 and 1.3. From the late 1990s to 2010, fluctuations intensified, but values were generally low, around 0.8 to 1.5. From 2010 to 2023, it was dominated by low values of 0.6 to 1.2, with intermittent short-term variations. The b-value rose to 0.45~0.75 between 1970 and 1980, then decreased and remained at 0.5~0.6 from 1980 to 1990. After 1990, it fluctuated dramatically, ranging from 0.48 to 0.8.

Region IX, which covers Taiwan, is affected by frequent strong earthquakes, and as a result, $M_{c}$ variations are particularly dynamic. From 1970 to 1987, the $M_{c}$ was approximately 1.0–2.0. It rose to 1.2–2.2 between 1987 and 1993, then decreased to 0.9–1.4 from 1994 to 2011. Since 2011, the $M_{c}$ has remained at a low level, stabilizing near 0.3. The b-value in this area showed large-amplitude fluctuations between approximately 0.35 and 0.7 from 1970 to 1990. From 1990 to 2000, these fluctuations diminished, and the value remained between 0.4 and 0.6. After 2000, fluctuation amplitudes increased again, reaching a maximum of 0.7.

### 4.2. Analysis of the Spatial Distribution of the $M_{c}$ and b-Values

This study employs a grid-based spatial statistics approach to analyze the spatial distribution characteristics of the $M_{c}$ and b-values. Specifically, the study area was first divided into a regular 0.1° × 0.1° grid. Then, centered at each grid node, a circular statistical unit with a 70-km radius was established. Seismic events within this radius were sampled to calculate the corresponding seismicity parameters, and the resulting values were assigned to that grid node.

To conduct an in-depth analysis of spatial variations in the $M_{c}$ and b-values, we divided the earthquake catalog into four temporal stages: 1970–1989, 1989–2001, 2001–2009, and 2009–2024. For each period, the same spatial scanning strategy was applied, but the number of seismic events used to calculate the $M_{c}$ and b-values was strictly constrained to be between 50 and 500 to ensure statistical results reliability. This period-specific spatial scanning approach effectively reveals the spatial heterogeneity in the $M_{c}$ and b-values distribution over different epochs.

**Figure 11:**
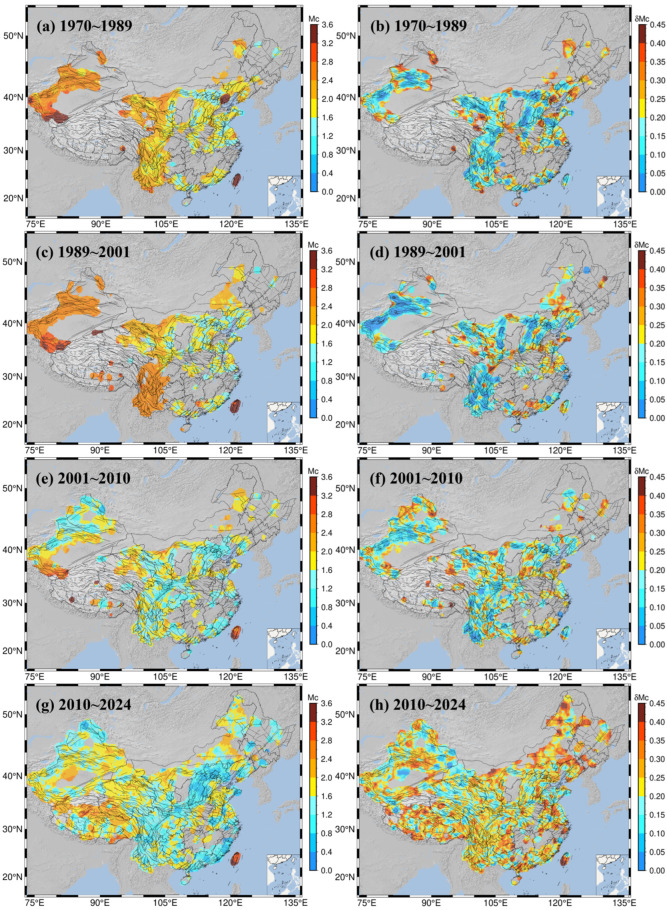
Spatial distribution of $M_{c}$ and ${\delta M}_{c}$ values in the study area for different time periods. (**a**,**c**,**e**,**g**) show $M_{c}$ values for 1970~1989, 1989~2001, 2001~2010, and 2010~2024, respectively; (**b**,**d**,**f**,**h**) show ${\delta M}_{c}$ values for the corresponding time periods. The color scales indicate $M_{c}$ values ranging from 0 to 3.6 and ${\delta M}_{c}$ values ranging from 0 to 0.45.

The calculated spatial distributions of the $M_{c}$ for the four time periods across mainland China are shown in Figure 11, where gray-white areas indicate regions with an insufficient number of events for calculation. During the 1970–1989 period, $M_{c}$ distribution in northwestern China was primarily concentrated along the Tianshan mountain range in Xinjiang. For instance, in the Western Kunlun belt of the Tibet–Xiyu block (western section), the $M_{c}$ ranged from 2.8 to 3.6, with a ${\delta M}_{c}$ between 0.1 and 0.25. Meanwhile, the $M_{c}$ in the North Tianshan belt was slightly lower than in the South Tianshan belt, at approximately 1.6~2.4, with a ${\delta M}_{c}$ not exceeding 0.1. The Haiyuan–Qilian belt, located in the Tibet–Xiyu block (eastern section), exhibited $M_{c}$ values between 2.0 and 3.2. In central mainland China, the $M_{c}$ was concentrated around the Ordos block in parts of Gansu and Ningxia, as well as in the Beijing–Tianjin–Tangshan area, Shanxi, and surrounding regions of North China. West of the Ordos block, the Helan Shan belt (part of the North China–Xiyu block) had an $M_{c}$ of 0.5~1.8. To the north, the Yinshan belt (part of the North China–Northeast Asia block, western section) showed an $M_{c}$ of 0.5~1.2, with a ${\delta M}_{c}$ of 0.05~0.25. To the south, the Qinling–Dabie Shan belt (part of the North China-South China block, eastern section) had an $M_{c}$ between 1.5 and 2.4. To the east, the Fen–Wei belt (part of the Ordos-North China Plain block) had an $M_{c}$ of 0.6–1.6, with a ${\delta M}_{c}$ below 0.1. East of the Fen–Wei belt are the Anyang–Heze–Linyi and Hebei Plain belts, where the $M_{c}$ ranged from 0.6 to 2.5, averaging around 1.5. The adjacent Yanshan–Bohai belt to the northeast (part of the North China–Northeast Asia block, eastern section) showed a high $M_{c}$ of 2.8~3.4 and a ${\delta M}_{c}$ of 0.35~0.4. In the southwest, the Minshan–Longmen Shan belt (northern section of the Tibet–South China block) and the Anninghe–Xiaojiang belt (southern section) had an $M_{c}$ between 1.6 and 2.6. The Red River belt had an $M_{c}$ of 2.0~3.2, while the Lancangjiang fault zone in the West Yunnan–South Yunnan block showed an $M_{c}$ of 2.0~3.4. Additionally, in the southeastern coastal regions of Fujian and Guangdong, influenced by seismic activity in Taiwan, the $M_{c}$ was between 1.1 and 2.4. Taiwan itself exhibited an $M_{c}$ above 3.6. In contrast, large parts of the northeast and the middle and lower reaches of the Yangtze River plain, central and eastern Inner Mongolia Plateau, and most of South China were predominantly gray areas.

During the 1989–2000 period, a polarized pattern emerged for $M_{c}$ along the Tianshan mountain range in Xinjiang. The $M_{c}$ in the Tianshan belt varied between 2.4 and 2.8, whereas the Western Kunlun belt showed a higher $M_{c}$ of 2.8–3.2; the ${\delta M}_{c}$ in both areas was below 0.1. The $M_{c}$ in the area between the Helan Shan and Haiyuan–Qilian belts decreased to 1.2~2.4. During this period, the $M_{c}$ in the southwestern region showed larger variations but was generally between 2.6 and 2.8, with a ${\delta M}_{c}$ below 0.05. The $M_{c}$ in the Yanshan–Bohai belt dropped to around 1.5. The $M_{c}$ in the coastal regions of Guangdong and Fujian decreased slightly, with an overall range of 1.0~2.0. The Anyang–Heze–Linyi and Hebei Plain belts showed elevated ${\delta M}_{c}$ values between 0.15 and 0.35. In Taiwan, the $M_{c}$ decreased to 3.0~3.8, with a ${\delta M}_{c}$ below 0.2.

From 2000 to 2009, the $M_{c}$ decreased substantially in regions such as the Tianshan belt, the Yanshan–Bohai belt, the Sichuan–Yunnan region, and the Guangdong–Fujian coast, falling to between 0.8 and 1.8. The $M_{c}$ in the Western Kunlun belt decreased to 2.0~2.8. After 2009, there was a significant nationwide decrease in the $M_{c}$, resulting in a more uniform spatial distribution. Additionally, seismic monitoring capabilities on the Tibetan Plateau block gradually improved after 2009, with $M_{c}$ values ranging from 1.0 to 3.0. Notably, the Himalayan belt on the southern margin of the Lhasa block and the Karakoram–Jiali belt of the Lhasa–Qiangtang block both showed $M_{c}$ values between 1.0 and 1.5. However, ${\delta M}_{c}$ spatial distribution during this period was highly heterogeneous, with values between 0.3 and 0.4 in areas such as the Tibetan Plateau, central Inner Mongolia, and the northeast. Even in the Chuandian experimental area, which has the densest station coverage, the ${\delta M}_{c}$ ranged from 0.1 to 0.35.

**Figure 12:**
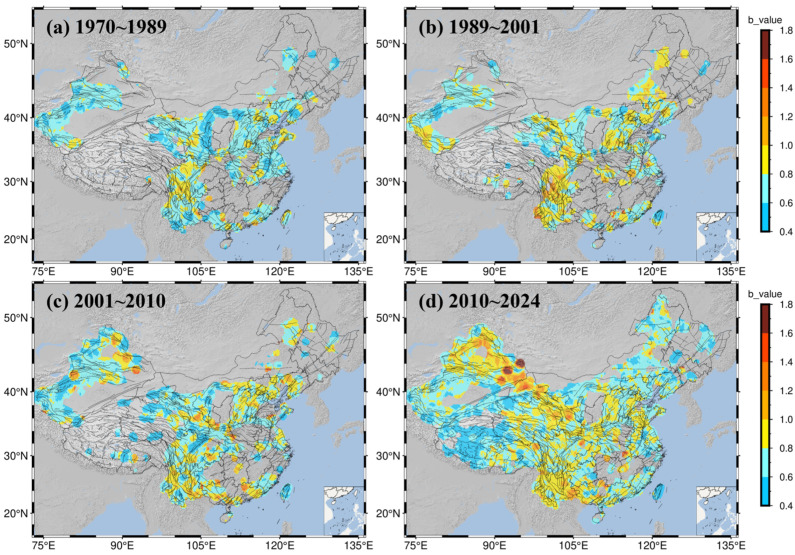
Spatial distribution of b-values in the study area for different time periods. (**a**) 1970~1989, (**b**) 1989~2001, (**c**) 2001~2010, (**d**) 2010~2024. The color scale indicates b-values ranging from 0.4 to 1.8.

The b-value results, calculated using the maximum likelihood method (Figure 12), show that the distribution of b-values exhibits significant variations across different time periods and regions.

Between 1970 and 1989, b-values exceeded 1.0 in only a few regions. Specifically, in the Helan Shan belt, South Tianshan belt, West Qinling–Delingha belt, the southern segment of the Red River belt, the Yinshan belt, and the middle segment of the Tan–Lu belt, b-values were primarily concentrated between 0.4 and 0.6. The b-value was between 0.6 and 0.8 in the Fen–Wei belt and areas to its west. In most of the Sichuan region, b-values were between 0.8 and 1.0, while the Yunnan region showed values between 0.6 and 0.8. In Taiwan, values were mostly distributed between 0.6 and 0.8, while the northern part showed values between 0.8 and 1.0.

During the 1989–2001 period, there was an overall increase in b-values across mainland China. Values rose to 0.8–1.0 in the Helan Shan and West Qinling–Delingha belts. In the Sanjiang belt, the western part of the Lancangjiang belt, the northern segment of the Red River belt, the junction of the Xianshuihe and Longmen Shan belts, and some areas in the southwest, b-values reached 1.0–1.4. The South Tianshan belt still maintained localized areas with values of 0.4–0.6, while the North Tianshan belt showed b-values of 0.6–0.8. The Western Kunlun belt reached b-values of 0.8–1.0. Taiwan exhibited a north–south contrast, with the northern part showing 0.4–0.6 and the southern part 0.8–1.0. Additionally, a localized zone of high b-values (1.2–1.4) appeared in northern Guangdong. Localized areas in the northeast also had b-values of 0.8–1.0.

From 2001 to 2010, b-values in the Tianshan belt were generally stable. Values in the central parts of the North and South Tianshan belts rose to 1.0–1.4, and high-value distributions also appeared in localized parts of the Fuyun belt. In the Chuandian (Sichuan–Yunnan) region, b-values generally decreased; however, localized areas such as the intersection of the Anninghe–Xiaojiang and Red River belts, the Longmen Shan fault zone, and the Lancangjiang belt maintained values of between 0.8 and 1.2. The Ordos block displayed a north–south difference: the western section of the Qinling–Dabie Shan belt in the south had b-values of 0.8–1.2, while values in the west and in the Helan Shan belt were generally lower than those in the Fen–Wei belt to the east. The b-value between the Helan Shan and Haiyuan–Qilian belts was 0.8–1.2. In South China, high values of 1.2–1.4 emerged in southern Guangxi and northern Guangdong, while western Guangxi and southern Guangdong showed values of 0.6–1.0. The South China Sea and central Taiwan were dominated by values of 0.4–0.6, while surrounding areas showed values of between 0.6 and 0.8.

Between 2010 and 2024, b-value distribution became more uniform, with most regions falling within the 0.8–1.0 range. Specifically, the western segment of the Haiyuan–Qilian belt in the northwest was 1.0–1.2, and the Fuyun belt of the Junggar–Altai block largely showed values of 1.0–1.4, with some localized spots exceeding 1.6. In contrast, the western Himalayan belt and the western Karakoram–Jiali belt on the Tibetan Plateau were notably lower, at only 0.4–0.6. Eastern regions of China generally had values of 0.6–1.0. The Chuandian region was primarily 0.8–1.0, though the area west of the intersection of the Red River and Xiaojiang fault zones reached 1.0–1.2. The South China Sea and Taiwan region generally had lower values, at only 0.4–0.6. The b-value distribution in other parts of South China, as well as the North China Plain and the northeastern regions, was relatively uniform, mostly ranging from 0.8 to 1.0.

## 5. Discussion and Conclusions

This study integrates multi-source earthquake catalogs and employs both partitioned time-series analysis and a gridded spatial scanning method to investigate the spatiotemporal evolution of the magnitude of completeness ($M_{c}$) and the b-value across mainland China and adjacent regions. The results reveal that variations in $M_{c}$ and the b-value not only exhibit significant temporal differences and spatial heterogeneity but are also controlled by the combined effects of tectonic environment, regional seismicity, and strong earthquake triggering.

Temporally, all nine seismic zones display phased evolution. Overall, the $M_{c}$ shows a clear trend from high, unstable values in the early period to low, stable values in recent decades. This reflects a substantial leap in monitoring capabilities, driven by the initial digitalization of the China National Seismic Network in the 1980s, expansive construction of national and regional digital networks around 2000, and the continuous station densification and technological upgrades in the 21st century.

However, major earthquakes can still trigger significant short-term rebounds in $M_{c}$. For instance, the 1976 Tangshan, 2008 Wenchuan, and the recent Luding and Maduo earthquakes all led to notable post-seismic increase in regional $M_{c}$ that persisted for some time, demonstrating the sensitive response of $M_{c}$ to strong seismic activity. In contrast, b-values are characterized by more pronounced volatility. Their episodic increases or decreases may reflect not only stress field and cyclical changes in fault activity but may also be influenced by catalog completeness evolution and monitoring conditions. Regionally, strong earthquake triggering effects are particularly significant in some areas (e.g., Sichuan–Yunnan, Taiwan, North China), where the b-value often shows abrupt changes or sharp fluctuations. In other regions (e.g., central and western Tibetan Plateau), they remain persistently low, implying a high stress accumulation in a seismogenic environment.

Spatially, both the $M_{c}$ and the b-value exhibit striking heterogeneity. In the 1970s and 1980s, under conditions of the early, relatively sparse analog network, the $M_{c}$ was generally high in the northwest and Taiwan, while b-values were broadly low, reflecting a catalog dominated by larger earthquakes. During the 1989–2001 period, with digitalization advancements and the initial establishment of the “China Digital Seismological Network” around the turn of the century, monitoring capabilities improved significantly. This led to an increased proportion of smaller earthquakes and the emergence of high b-value zones along some major tectonic belts (e.g., the Lancangjiang, Red River, and Longmen Shan fault zones). From 2001 to 2009, as projects like the “China Digital Seismic Observation Network” were fully implemented, nationwide $M_{c}$ decreased, but low b-value anomalies emerged at several fault intersections, indicating stress concentration. Since 2009, the $M_{c}$ has trended toward low and uniform values, while b-values have generally fluctuated between 0.8 and 1.0. A stark contrast has formed: stable blocks in the northwest show b-values commonly above 1.0 (some exceeding 1.6), whereas the western Tibetan Plateau and the Himalayan–Karakoram belt maintain low levels of 0.2~0.6. This spatial gradient reflects fundamental differences in seismogenic mechanisms under different tectonic settings: stable blocks are dominated by small-magnitude seismicity and more uniform rupture processes, whereas highly deformed zones are characterized by stress concentration and more significant contribution from strong earthquakes.

In summary, the long-term decrease and stabilization of $M_{c}$ are primarily attributable to improvement observational capability and catalog completeness resulting from decades of continuous network development—from digitalization and networking to high-density deployment. Its episodic rebounds, however, are closely linked to the triggering effects of strong earthquakes. The b-value fluctuations reveal regional seismogenic environments complexity, where low b-values often correspond to zones of stress concentration or areas dominated by large earthquakes, and high b-values indicate an increased proportion of small earthquakes or trend toward more homogeneous medium fracture. This characteristic is, on the one hand, related to strong earthquake triggering and aftershock sequences, as major events often cause the b-value to drop or rise abruptly in the short term. On the other hand, it may also reflect stress field redistribution and the evolution of rupture patterns. For example, the b-value in the Sichuan–Yunnan region dropped sharply during the Wenchuan earthquake and subsequently recovered to a higher level, illustrating the dynamic adjustment of regional stress accumulation and release. Furthermore, persistently low b-values in the western Tibetan Plateau suggests that its crust is in a state of high stress accumulation, implying higher potential seismic risk. Conversely, high b-values in the stable blocks of the northwest suggest that stress is primarily released through small earthquakes within a relatively homogeneous seismogenic environment.

In this study, the b-value was calculated within a spatiotemporal moving window using a dynamic, time-varying $M_{c}$, rather than a single, fixed value for the entire study period. Using a fixed $M_{c}$ would inevitably introduce systematic biases. For the early stages of China’s seismic network, a low fixed $M_{c}$ would erroneously include incomplete microseismic data, potentially leading to artificial depression of the b-value. Conversely, for the recent period of significantly improved monitoring, a high fixed $M_{c}$ would discard numerous of valid, well-recorded microearthquakes above the true $M_{c}$, which would impair the sensitivity of the b-value to subtle changes in the stress field. Therefore, the spatiotemporal b-value evolution derived from our method effectively decouples the systematic $M_{c}$ decrease caused by network upgrades from b-value fluctuations caused by changes in stress state. This ensures that b-value evolution analysis genuinely reflects tectonic stress changes rather than artifacts of the evolving observation system.

Earthquake prediction models rely on arrays of seismicity parameters derived from earthquake catalogs. These metrics include the time to the nth event, average magnitude, the difference between observed and expected magnitudes, the Gutenberg–Richter slope (b-value), mean square deviation, the square root of released energy, and mean inter-event time, among others; the microseismicity presence also influences large earthquake forecasts. Consequently, any incompleteness or bias in these catalogs will directly impact the feature engineering process, thereby degrading model performance. Furthermore, the spatiotemporally evolving b-value, which encodes information about the regional stress states, can be directly incorporated into forecasting models as a key physical feature.

Notably, our characterization of b-value variations remains primarily descriptive. Although we have explored the consistency between observed patterns and known tectonic settings, quantitative comparisons with independent geophysical parameters—such as strain rate, fault slip rate, lithospheric temperature, or Coulomb stress changes—would provide more robust constraints on potential physical mechanisms. Second, our analysis’s spatial resolution is limited by the sample size required for statistically reliable b-value estimation. Despite employing a spatiotemporal variable $M_{c}$ approach, uncertainties in determining $M_{c}$ during network transition periods or postseismic intervals may still introduce systematic biases in b-value calculations. Third, while we have identified regional-scale b-value patterns, distinguishing whether observed anomalies (such as pre-seismic b-value decreases) reflect genuine stress accumulation or catalog artifacts requires complementary analyses such as focal mechanism studies and stress field inversions. Finally, translating these regional findings into operational earthquake forecasts necessitates rigorous validation through high-resolution, fault-specific, retrospective analyses. Future work requires collaborative research that integrates multi-parameter geophysical observations and focuses on key seismogenic fault zones to establish more direct linkages between b-value evolution and earthquake preparation processes.

This research has revealed macro-scale features, such as the long-term low b-value signature in large areas like the western Tibetan Plateau. However, strong earthquake preparation and occurrence is ultimately a localized physical process. High-resolution, retrospective spatiotemporal b-value scanning should be conducted on specific fault zones with strong earthquakes history (e.g., the Longmen Shan fault zone, the Xianshuihe fault zone). Such studies could test for any systematic and recognizable precursory b-value decrease in the epicentral area and adjacent fault segments in the years or months leading up to a mainshock. For these sequence-scale analyses, the b-positive family of estimators introduced by van der Elst and refined by Lippiello and Petrillo offer particular advantages. Unlike the regional-scale MLE approach employed in this study, these increment-based methods can circumvent the challenge of determining time-varying $M_{c}$ during chaotic postseismic periods and provide more efficient utilization of transiently incomplete catalog segments characteristic of active aftershock sequences. Applying these advanced estimators to major sequences identified in our regional analysis—such as the 2008 Wenchuan, 2013 Lushan, or 2021 Maduo earthquakes—would enable robust b-value monitoring throughout all earthquake evolution phases, from foreshock activity through the mainshock to late-stage aftershock decay. Such high-resolution, sequence-specific investigations would complement our regional findings by revealing fine-scale stress evolution patterns that may be obscured in spatiotemporally aggregated catalogs. This is critically important for exploring short-term and imminent earthquake precursors. Therefore, future research should shift from macro-scale analysis to a micro-scale focus on key fault zones and specific seismogenic segments, integrating complementary estimation methodologies optimized for different spatiotemporal scales to construct more comprehensive understanding of the earthquake preparation process.

## Figures and Tables

**Table 1:** Characteristics of the active block boundaries in mainland China (from Zhang et al., 2005) [38].

No.	Name	Names of Flanking Blocks
1	Yanshan–Bohai Belt	North China Craton—Northeast Asia (Eastern Segment)
2	Yinshan Belt	North China Craton—Northeast Asia (Western Segment)
3	Helanshan Tectonic Belt	North China Craton—Western Regions Block
4	Minshan–Longmenshan Belt	Qinghai–Tibet Block—South China Block (Northern Segment)
5	Anninghe–Xiaojiang Belt	Qinghai–Tibet Block—South China Block (Southern Segment)
6	Red River Belt	Qinghai–Tibet Block—Sibumasu Terrane
7	Haiyuan–Qilian Belt	Qinghai–Tibet Block—Western Regions Block (Eastern Segment)
8	Altyn Tagh Belt	Qinghai–Tibet Block—Western Regions Block (Central Segment)
9	West Kunlun Belt	Qinghai–Tibet Block—Western Regions Block (Western Segment)
10	Fen–Wei Belt	Ordos Block—North China Plain
11	Qinling–Dabie Belt	North China Craton—South China Block (Eastern Segment)
12	Tan-Lu Belt	North China Plain—East Shandong–Yellow Sea Block
13	Southeast Coastal Belt	South China Block—South China Sea Block
14	Himalayan Belt	Southern Margin of the Lhasa Terrane
15	Karakoram–Jiali Belt	Lhasa Terrane—Qiangtang Terrane
16	Maini–Yushu Belt	Qiangtang Terrane—Bayan Har Block
17	Sanjiang Belt	Qiangtang Terrane—Sichuan–Yunnan Block
18	Xianshuihe Belt	Bayan Har Block—Sichuan–Yunnan Block
19	East Kunlun Belt	Bayan Har Block—Qaidam Basin
20	West Qinling–Delingha Belt	Qilian Block—Qaidam Basin
21	South Tianshan Belt	Tarim Basin—Tianshan Block
22	North Tianshan Belt	Tianshan Block—Junggar Basin
23	Fuyun Belt	Junggar Basin—Altai Block
24	Lancangjiang Belt	West Yunnan Block—South Yunnan Block
25	Hebei Plain Belt	Tertiary Block Boundary
26	Anyang–Heze–Linyi Belt	Tertiary Block Boundary

**Table 2:** Regional divisions and associated active block boundary zones.

No.	Included Belts	Names of Flanking Blocks	Geographic Range (Lon/Lat)
I	South Tianshan Belt, North Tianshan Belt, Fuyun Belt	Tarim—Tianshan—Junggar—Altai	73.5°~97.5° E; 39.8°~49.4° N
II	West Kunlun Belt, Altyn Tagh Belt	Qinghai–Tibet Block—Western Regions Block (Western and Central Segments)	73.5°~97.5° E; 36.0°~39.8° N
III	Himalayan Belt, Karakoram–Jiali Belt	Southern Margin of the Lhasa Terrane, Lhasa Terrane—Qiangtang Terrane	75.8°~97.5° E; 27.5°~36.0° N
IV	West Kunlun Belt, Altyn Tagh Belt, Maini-Yushu Belt, Sanjiang Belt, Xianshuihe Belt, East Kunlun Belt, West Qinling–Delingha Belt	Qinghai–Tibet Block—Western Regions Block (Western and Central Segments), Qiangtang Terrane—Chuandian Block, Bayan Har Block—Qaidam Basin	97.55°~106.0° E; 33.0°~42.0° N
V	Minshan–Longmenshan Belt, Anninghe–Xiaojiang Belt, Red River Belt, Lancangjiang Belt	Qinghai–Tibet Block—South China Block (Northern and Southern Segments), Sibumasu Terrane, West Yunnan Block—South Yunnan Block	97.5°~106.0° E; 21.0°~33.0° N
VI	Yanshan-Bohai Belt	North China Craton—Northeast Asia (Eastern Segment)	106.0°~135.0° E; 41.4°~53.0° N
VII	Yanshan–Bohai Belt, Fen–Wei Belt, Qinling–Dabie Belt	North China Craton—Northeast Asia (Eastern Segment), Ordos Block—North China Plain, North China Craton—South China Block (Eastern Segment)	106.0°~123.5° E; 35.0°~41.4° N
VIII	Qinling–Dabie Belt, Tan–Lu Belt	North China Craton—South China Block (Eastern Segment), North China Plain—East Shandong–Yellow Sea Block	106.0°~123.5° E; 29.7°~35.0° N
IX	Southeast Coastal Belt	South China Block—South China Sea Block	106.0°~123.5° E; 18.0°~29.7° N

## Data Availability

The earthquake catalog used in this study was provided by the China Earthquake Networks Center (CENC) and the Second Monitoring Center, China Earthquake Administration.
